# How the histological structure of some lung cancers shaped almost 70 years of radiobiology

**DOI:** 10.1038/s41416-022-02041-9

**Published:** 2022-11-07

**Authors:** Katja R. Worth, Ioanna Papandreou, Ester M. Hammond

**Affiliations:** 1 Oxford Institute for Radiation Oncology, Department of Oncology, University of Oxford, Old Road Campus Research Building, Oxford, OX3 7DQ UK; 2 Department of Radiation Oncology, OSUCCC and Wexner Medical Center, The Ohio State University, Columbus, OH 43210 USA

**Keywords:** Cancer microenvironment, Cancer therapy

## Abstract

Pivotal research led by Louis Harold Gray in the 1950s suggested that oxygen plays a vital role during radiotherapy. By proving that tumours have large necrotic cores due to hypoxia and that hypoxic cells require significantly larger doses of ionising radiation to achieve the same cell kill, Thomlinson and Gray inspired the subsequent decades of research into better defining the mechanistic role of molecular oxygen at the time of radiation. Ultimately, the work pioneered by Thomlinson and Gray led to numerous elegant studies which demonstrated that tumour hypoxia predicts for poor patient outcomes. Furthermore, this subsequently resulted in investigations into markers and measurement of hypoxia, as well as modification strategies. However, despite an abundance of pre-clinical data supporting hypoxia-targeted treatments, there is limited widespread application of hypoxia-targeted therapies routinely used in clinical practice. Significant contributing factors underpinning disappointing clinical trial results include the use of model systems which are more hypoxic than human tumours and a failure to stratify patients based on levels of hypoxia. However, translating the original findings of Thomlinson and Gray remains a research priority with the potential to significantly improve patient outcomes and specifically those receiving radiotherapy.

## Introduction

The origins of radiotherapy date back to 1896 when Emil Herman Grubbé utilised X-rays, less than 60 days after their discovery by Wilhelm Conrad Röntgen, to treat a case of advanced ulcerative breast cancer in Chicago with little knowledge of their physical properties or biological effects [1]. Over successive decades, rigorous scientific research was conducted to facilitate the continuous advancements in knowledge of the fundamental mechanisms of action and factors affecting radiosensitivity. One important name underlying a significant proportion of research during this time is Louis Harold Gray, the British physicist whose landmark contributions inspired the naming of the SI unit of absorbed ionising radiation dose. In the 1950s, Gray began studying the importance of tumour oxygen concentration and hypoxia in the context of radiotherapy efficacy with colleagues, revealing critical findings that provided solid foundations for the years of radiobiological research that have followed [2, 3].

**Fig. 1:**
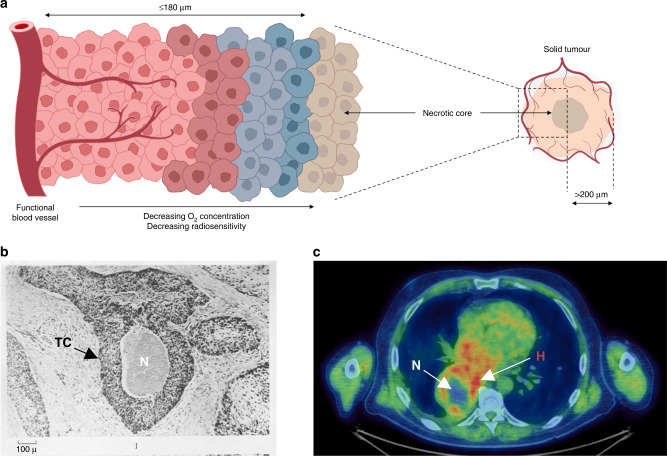
Regions of hypoxia (insufficient oxygen) lead to necrosis in solid tumours. **a** Schematic illustration summarising the key findings from Thomlinson and Gray [3]. In solid tumours, cells proximal to blood vessels are well oxygenated, whilst cells at a distance become increasingly hypoxic with increased distance from the functional blood supply. Necrosis was found to occur ≤180 µm from the blood supply, resulting in necrotic cores at the centre of solid tumours with a total radius of >200 µm [3]. The figure includes adapted templates from Servier Medical Art (with permission). **b** Transverse section of tumour chord (labelled ‘TC’) from carcinoma of the bronchus demonstrating that cords greater than 180 μM have necrotic centres (labelled ‘N’). Image reproduced from Thomlinson and Gray [3]. **c** Axial image of F-MISO PET CT scan showing large NSCLC with hypoxic region (red, labelled ‘H’) surrounding a necrotic core (blue, labelled ‘N’) without F-MISO penetration. Image supplied by Dr Daniel McGowan and Dr. Geoff Higgins (University of Oxford).

In 1955, Gray published a research paper in collaboration with Raymond Hugh Thomlinson titled “The histological structure of some human lung cancers and the possible implications for radiotherapy” in the British Journal of Cancer [3]. At this time, it had been established that tumours contained hypoxic regions and the levels of oxygenation could determine radiosensitivity and tumour regression [4, 5]. By cutting histological sections of human epithelial tumours, Thomlinson and Gray observed areas of necrosis in the centre of large tumours which were encompassed by rings of intact tumour cells [3]. More specifically, in 159 out of the 160 tumour areas measured and studied, the authors were able to determine that central tumour necrosis was absent in all tumour sections <160 µm in radius whilst present in all tumour sections >200 µm in radius [3]. In addition, they established that the radius of intact tumour cells that surrounded the central necrotic core never exceeded 180 µm; instead, the radius of the necrotic core increased as the overall tumour size increased [3]. As illustrated in Fig. 1a, b, Thomlinson and Gray suggested that these observations were due to a decreasing gradient in oxygen tension from the periphery to the tumour centre, the magnitude of which is dependent on oxygen consumption, since the blood vessels supplying oxygen were identified toward the tumour epithelial surface but not intertwined deep into the tumour bulk [3]. In conclusion, Thomlinson and Gray identified necrosis as one of the first distinguishable biomarkers of tumour hypoxia.

## Implications for radiotherapy

These seminal findings from Thomlinson and Gray in 1955 built upon the prior pivotal research of Gray et al., which demonstrated that tumour cells experiencing anoxia at the time of irradiation are ~2.5–3 times more radioresistant to a given dose of X-rays or γ-rays than oxygenated tumour cells [2]. Together, the results clearly demonstrated an oxygen concentration gradient that reduces from the periphery to the innermost part of the tumour sections, thus suggesting that the central tumour cells would be more radioresistant due to the presence of less molecular oxygen at the time of irradiation [2, 3].

In addition, Thomlinson and Gray noted that a given dose of radiation, sufficient to cause cell death in the outer radiosensitive cells, will increase the supply of oxygen and other nutrients to the central necrotic cells, allowing the cells to continue proliferation if they have retained the capacity to replicate following the period of oxygen deprivation [3]. Today, this reoxygenation of hypoxic cells that follows irradiation is known as one of the 5R’s (repair, repopulation, reoxygenation, redistribution and radiosensitivity) which are all supporting factors that provide the rationale for fractionation of clinical radiotherapy regimens [6].

At the time of this pivotal research, the influence of oxygen tension at the time of irradiation on the biological response to radiotherapy had not been mechanistically evaluated [2]. However, the widely accepted theory to explain this close relationship is now known as the oxygen fixation hypothesis [7]. During radiation treatment, incoming X-ray photons interact with surrounding water molecules in biological matter, in a reaction known as water radiolysis, which generates highly reactive hydroxyl radicals (OH•) capable of directly interacting with DNA and producing DNA radicals (DNA•) [7, 8]. In the presence of oxygen, reactions between DNA• and molecular oxygen yield a permanent DNA peroxyl radical (DNA–OO•) which is challenging or even impossible to repair [7, 8]. Hence, the damage is considered ‘fixed’ in place in oxygenated cells targeted with radiation. Conversely, in conditions of hypoxia, the lack of available oxygen permits DNA• radicals to be efficiently repaired via donation of hydrogen ions from cellular thiols which chemically restores the DNA structure [7, 8]. Today, a value known as the oxygen enhancement ratio (OER) is calculated to represent the ratio of radiation doses required under hypoxia to achieve the same cell kill levels as under normal physiological conditions. For X-rays and γ-rays this OER is 2.5–3, therefore emphasising that hypoxic tumours require significantly higher radiation doses to be controlled or cured [2, 7].

## Moving on from necrosis—markers of tumour hypoxia

As previously, the pioneering work of Thomlinson and Gray highlighted central cores of necrotic cells as the first indicator of tumour hypoxia. Necrosis of tumour cells occurs as a result of chronic or diffusion-limited hypoxia at distances >180 μM from functional blood vessels [3, 9]. As predicted by Thomlinson and Gray, a gradient in oxygen concentration occurs between the tumour vasculature and necrotic regions due to chronic or diffusion-limited hypoxia, which occurs due to cells closest to functional tumour vessels metabolising all available oxygen and leaving none for cells at the tumour core. This was validated and quantified in later years following the emergence of Eppendorf polarographic oxygen electrodes as a methodology for direct measurements of intratumoral oxygen tension [10, 11]. These oxygen probes are often referred to as the “gold standard” tool for direct tumour hypoxia determination and were frequently successful in predicting treatment outcome, however, their clinical use is limited today due to associated invasiveness [10, 11]. Importantly, the probes often overestimated hypoxic fractions since they are not able to distinguish necrotic tissue from viable hypoxic tissue [10, 11]. Although not currently widely used, necrosis remains an important marker of tumour hypoxia and predictor of those patients likely to benefit from hypoxia modification [12].

After Thomlinson and Gray highlighted that hypoxia could be visualised through necrosis, significant effort was invested in developing probes for hypoxia in both the laboratory and clinical settings. For example, the 2-nitroimidazole compounds Pimonidazole and EF5 act as predictive biomarkers for hypoxia by being reduced and covalently binding intracellular macromolecules specifically at low oxygen concentrations <10 mmHg [13, 14]. Pimonidazole and EF5 binding can be detected either immunohistochemically with antibodies against the formed macromolecule adducts, or non-invasively by positron emission tomography (PET) imaging, a type of imaging modality in nuclear medicine if labelled with radionuclides [14]. Subsequently, several redox-sensitive molecules, notably nitroimidazoles and Cu-chelated complexes, have been clinically tested as tracers and found to predict hypoxia and therapy outcomes [15]. However, factors such as slow pharmacokinetics and low tumour-to-background signal ratio, oxygen-independent efflux from cells, poor tumour penetration, as well as cost have limited their widespread applicability [16]. In contrast, ^18^F-fluoromisonidazole (FMISO) is one of several ^18^F-labelled tracers more recently utilised in oncology to specifically detect hypoxic tumour regions. Lipophilic FMISO diffuses into a cell where its nitro group is reversibly reduced via the addition of two electrons by nitroreductase enzymes [17]. In oxygenated cells, the reduced FMISO is re-oxidised and therefore can diffuse out of the viable cell where it circulates and is eventually excreted [17]. However, in hypoxic cells, the lack of available molecular oxygen prevents re-oxidation of the reduced FMISO molecule, thus causing its accumulation and retention in the intracellular space whereby it can be quantitatively imaged using PET [17] (Fig. 1c). The use of FMISO is beneficial since the dynamic range of retention falls within the range of radiobiological hypoxia (<0.1% O_2_) [18], allowing the signal intensity to create parametric maps that indicate the oxygen distribution within a tissue or tumour [19]. FMISO PET can be used to measure hypoxia predictively or prognostically, however, is not yet routinely used clinically to image hypoxia beyond the clinical trial setting [14].

Conversely, endogenous markers of tumour hypoxia are genes or proteins whose expression is selectively induced in conditions of hypoxia. The most studied are the hypoxia-inducible factors (HIFs), the master orchestrator of the biological response to physiological hypoxia, whose transcriptional activity is highly dependent on cellular oxygen concentration [18, 20]. As transcription factors, the HIF family regulate the expression of numerous genes involved in fundamental cellular processes that promote tumour cell survival in the hostile hypoxic tumour microenvironment. Some well-characterised genes that are directly transcriptionally activated in response to HIF signalling related to glucose metabolism, angiogenesis and pH homoeostasis include glucose transporters 1 and 3, pyruvate dehydrogenase kinases, vascular endothelial growth factor and carbonic anhydrase IX [18]. Using endogenous markers is advantageous as they can be detected in archived specimens, meaning no additional biopsies are required [14]. Despite their expression being significantly associated with poor prognosis and treatment outcomes, their use is limited by their poor hypoxia specificity since they are indirect functional biomarkers whose expression kinetics may only modestly correlate with hypoxia [21–23]. Interestingly, recent developments in robust hypoxic-specific gene expression signatures have demonstrated larger statistical power than any single endogenous biomarker alone, therefore increasing potential benefit as accurate predictive and prognostic markers more suited to applications in a clinical context [24].

More recently, it has become apparent that not only does hypoxia exist as a gradient, but the biological response to hypoxia differs in an oxygen-dependent manner [25, 26]. For example, whilst stabilisation of the HIFs occurs in relatively mild levels of hypoxia, responses, including the unfolded protein response or DNA damage response, are associated with more severe levels of hypoxia [25, 26]. Consequently, significant effort has been directed towards the identification and validation of novel exogenous and endogenous biomarkers to identify and assess tumour hypoxia in research today [14, 19].

## Clinical relevance of hypoxia

As a hallmark characteristic of solid tumours, the clinical importance of tumour hypoxia as a strong predictive biomarker indicating a likely poor response to both chemotherapy and radiotherapy treatments is well-established, with the latter being first revealed as significant by Gray et al. in 1953 [2]. In addition, subsequent research revealed that the presence of tumour hypoxia is strongly associated with poor patient outcomes and prognosis across a range of solid tumour types [27–30]. Hypoxia is associated with resistance to chemotherapy due to the chaotic and disorganised nature of tumour vasculature as a result of aberrant angiogenesis pathways, resulting in blood flow variations that significantly hinder effective drug delivery to all regions of the tumour [31]. Hypoxic cells are also able to evade therapy by undergoing adaptations, the majority of which result from HIF pathway activation and the consequent transcriptional upregulation, which confer a selective survival advantage in comparison to other tumour clones [32, 33]. Examples of such adaptations include increasing cell motility and driving metastasis through regulation of the epithelial-to-mesenchymal transition in addition to increased stemness [32, 33]. More recently, with the advent of immunotherapies, hypoxia has been linked to a phenomenon known as immune exclusion, thus limiting the efficacy of immune-oncology therapeutic agents [32]. Hypoxia-mediated immune exclusion is believed to occur due to physical barriers, such as vascular accessibility in the aberrant tumour vasculature, hypoxic cytokine-mediated immunosuppression involving adenosine production, cancer cell coating with the inhibitory chemokine CXCL12 to shield them from Tcells, and also a variety of functional barriers, such as nutrient depletion or other metabolic issues affecting T-cell function [34]. Taken together, Thomlinson and Gray were the first to suggest that the presence of hypoxia and its associated signalling are drivers of a highly aggressive tumour phenotype, which has been well-validated by subsequent research and emphasises an urgent clinical need for accurate hypoxia identification and modification in patient tumours.

Over the recent decades, several hypoxia modification strategies have been developed and investigated in an attempt to improve patient outcomes (reviewed by Horsman et al. [35]), however, most reported improvements in outcomes are modest due to the heterogeneity in hypoxia distribution, lack of hypoxia assessment and poor patient selection and stratification in clinical studies [14, 36]. One early approach aimed to increase oxygen delivery to tumours by allowing patients to breathe hyperbaric oxygen (100% oxygen) or carbogen (95% oxygen and 5% carbon dioxide). Carbogen was thought to influence chronic hypoxia but have no effect on acute hypoxia, occurring as a consequence of transient blood vessel flow changes, and so its benefit was limited unless in combination with the radiosensitiser nicotinamide [37, 38]. The Accelerated Radiation, CarbOgen and Nicotinamide (ARCON) trial clinically evaluated this combination treatment and reported comparable toxicity and positive improvements in regional control rate in head and neck cancers [39, 40]. The use of hyperthermia immediately following radiotherapy has also been effective in enhancing sensitisation and radiation response of hypoxic cells in a number of tumour types [41]. The radiosensitisation effect is only observed if there is no time interval between irradiation and the given heat treatment, as it is thought that the immediate effect of heat is what kills hypoxic cells [37]. In addition, targeting HIF signalling was explored as a potential therapeutic strategy, and several HIF inhibitors achieved pre-clinical success, but the complexity of HIF biology in combination with toxicities due to the non-specificity of some currently available inhibitors has made successful translation challenging [42]. Despite this, an oral inhibitor of HIF-2α (belzutifan) was recently FDA approved in 2021 for use in patients with Von Hippel–Lindau disease-associated cancers such as renal cell carcinoma following promising results from a Phase II trial (NCT03401788) [43].

In addition, the first proposed some decades ago was hypoxia modification through the development of hypoxia-activated prodrugs (HAPs). HAPs are compounds that are selectively reduced by reductase enzymes exclusively in regions of hypoxia to yield cytotoxic agents. Examples include tirapazamine [44, 45], TH-302 [46] and CP-506 [47]. HAPs are still thought to be a potential method for sensitising hypoxic tumour regions to radiotherapy and chemotherapy, despite the limited success so far due to unanswered questions regarding action and toxicity [44, 45, 48]. Recent developments in this area of research include the development of molecularly targeted HAPs [49, 50].

A more recent strategy to eliminate tumour hypoxia by correcting the imbalance between oxygen supply and demand is via decreased oxygen consumption resulting in increased availability of oxygen to hypoxic cells [51]. This strategy is based on theoretical models that have revealed that moderate changes in oxygen utilisation can significantly impact overall oxygenation [52]. For example, atovaquone is a previously FDA-approved drug known to inhibit complex III of the mitochondrial electron transport chain, thus reducing oxygen consumption without killing hypoxic cells [53]. Results from the ATOM clinical trial have revealed large reductions in tumour hypoxic volumes post-treatment with atovaquone, assessed using FMISO PET imaging, in non-small cell lung cancer (NSCLC) [54]. Currently, the ARCADIAN Phase I aims to establish the maximum tolerable dose of atovaquone in combination with radical chemoradiotherapy for patients with NSCLC (NCT04648033). Another repurposed drug being investigated as a hypoxic modulator is papaverine which has known phosphodiesterase 10A inhibitory activity but also inhibits mitochondrial complex I activity, and through this latter off-target activity is able to decrease hypoxia in vivo and radiosensitise tumour models [55, 56]. Phase I clinical testing of papaverine combined with either stereotactic body radiotherapy (NCT03824327) or chemoradiation (NCT05136846) for NSCLC is currently underway with drug-induced changes in oxygenation being assessed as a secondary endpoint.

## Conclusion

Today, tumour hypoxia is well-established as a critical characteristic of the majority of solid tumours and is closely associated with negative treatment outcomes in a clinical setting. Data generated in the 1950s, including that published by Thomlinson and Gray, suggested a critical role of oxygen in radiation therapy efficacy and identified regions of necrosis as a consequence of tumour hypoxia. Importantly, both of these key findings have since been mechanistically validated.

Inspired by this pivotal research, extensive studies have been undertaken to better define hypoxic regions, develop measurement and quantification methods, as well as propose targeted modification strategies [18]. Issues related to appropriate methodology and patient selection in previous clinical trials have limited the successful translation of hypoxia-specific therapies thus far. However, the interest and abundance of pre-clinical research in support of hypoxia-modifying treatments in combination with improved hypoxia measurement strategies encourage future clinical trials. The ability to translate research from 75 years ago through the introduction of personalised, hypoxia-specific therapeutic strategies in combination with accurate tumour hypoxia assessment remains an important and unrealised goal to advance cancer therapy. Successful translation has the potential to yield meaningful therapeutic benefits and improve individual patient outcomes, specifically for the multitude of patients that receive radiotherapy as part of their treatment regimens.
